# Changing distribution of age, clinical severity, and genotypes of rotavirus gastroenteritis in hospitalized children after the introduction of vaccination: a single center study in Seoul between 2011 and 2014

**DOI:** 10.1186/s12879-016-1623-y

**Published:** 2016-06-14

**Authors:** Jung Ok Shim, Ju Young Chang, Sue Shin, Jin Soo Moon, Jae Sung Ko

**Affiliations:** Department of Pediatrics, Korea University College of Medicine, 148, Gurodong-ro, Guro-gu, Seoul 152-703 South Korea; Department of Pediatrics, Seoul National University College of Medicine, 101, Daehak-ro, Jongno-gu, Seoul 110-744 South Korea; Seoul Metropolitan Government Seoul National University Boramae Medical Center Seoul, 20, Boramae-ro 5-gil, Dongjak-gu, Seoul 156-707 South Korea; Department of Laboratory Medicine, Seoul National University College of Medicine, 101, Daehak-ro, Jongno-gu, Seoul 110-744 South Korea

**Keywords:** Rotavirus, Genotype, Age, Clinical severity, Vaccine

## Abstract

**Background:**

This study aimed to explore changes in clinical epidemiology and genotype distribution and their association among hospitalized children with rotavirus gastroenteritis after the introduction of vaccines.

**Methods:**

Between November 2010 and October 2014, hospitalized children with acute gastroenteritis were enrolled. Rotavirus genotypes were confirmed through reverse transcription-polymerase chain reaction (RT-PCR), semi-nested PCR, and sequencing. Clinical information including vaccination status and the modified Vesikari scores were collected.

**Results:**

Among 179 children with rotavirus infection, nineteen (10.6 %) were completely vaccinated. During the study period, the number of children between three and 23 months of age decreased significantly compared to the number of children older than 24 months of age (*P* = 0.010), who showed lower diarrhea severity (duration, *P* = 0.042; frequency, *P* = 0.021) but higher vomiting severity (*P* = 0.007, 0.036) compared to the former. Vaccination status was also significantly associated with lower vomiting severity after adjustment for age (frequency only, *P* = 0.018). The predominant genotypes were G2P[4] (18.4 %), G1P[8] (14.5 %), and G1P[4]P[8] (12.8 %), and the prevalence of genotypes with uncommon and mixed combinations was more than 50 %. Children infected with G2P[4] strains tended to be older (*P* = 0.005) and had more severe vomiting (*P* = 0.018, 0.006) than those with G1P[8].

**Conclusions:**

Increase in age of infected, hospitalized children was accompanied by change in clinical severity during 2011–2014 after the introduction of vaccines in Seoul. Clinical severity was also associated with vaccination status and genotype. Long-term large scale studies are needed to document the significance of the increase in genotypes of uncommon and mixed combinations.

**Electronic supplementary material:**

The online version of this article (doi:10.1186/s12879-016-1623-y) contains supplementary material, which is available to authorized users.

## Background

Currently, rotavirus vaccination plays a key role in protecting children against rotavirus-related hospitalizations in countries implementing rotavirus vaccination [1–3]. In Korea, two rotavirus vaccines, RotaTeq (Merck and Company, Whitehouse Station, NJ, USA) and Rotarix (GlaxoSmithKline Biologicals, Rixensart, Belgium) were introduced in 2007 and 2008, respectively [1, 4]. Although rotavirus vaccines are recommended but not compulsory, the rate of individuals who had received two or three doses of a rotavirus vaccine was reported to be 65.6 % in Seoul and 52.4 % nationwide (2013 Korean National Immunization Survey, http://www.cdc.go.kr). As a result, the proportion of rotavirus infection among hospitalized children with gastroenteritis has been decreasing remarkably [2, 5]. According to a recent Korean Center for Disease Control report (http://www.cdc.go.kr), the incidence of rotavirus gastroenteritis was lower than that of norovirus in 2015. The effectiveness of vaccination against rotavirus-related hospitalization was suggested in six Asian countries including Korea [6]. However, rotavirus gastroenteritis is still a major cause of illness requiring hospitalization even among rotavirus-vaccinated children. In addition, there is still debate on whether diversity of rotavirus strains contributes to low vaccine efficacy [7, 8]. Thus, in addition to efforts to increase vaccination rates, ongoing surveillance of rotavirus genotypes is necessary, particularly in children with vaccine failure [9].

Regarding the other notable changes in clinical epidemiology after the introduction of rotavirus vaccines, a few studies reported patterns of seasonal change or age change in addition to decreased overall prevalence of rotavirus infection [2, 5, 10]. A delay of the rotavirus season by 2–3 months was observed in Seoul in a multicenter study during 2007–2010 [2]. A few single-center studies in the Korean literature reported that the age of children with rotavirus infection in Seoul or its suburbs increased significantly between 2008 and 2013 compared to the pre-vaccine era [5, 10]. It is unclear whether these changes have been consistent and ongoing. Before the introduction of rotavirus vaccines, rotavirus gastroenteritis usually occurred in children less than 24 months of age and presented with the most severe clinical manifestations including diarrhea among all types of viral gastroenteritis [11]. Because severe diarrhea is usually believed to be prevalent in younger children [11], the shift of infected age to rotavirus infection might be accompanied by changes in clinical manifestation such as diarrheal severity of rotavirus gastroenteritis. In addition, although debatable, clinical factors such as age and severity of gastroenteritis have also been associated with certain genotypes according to several studies from the pre-vaccine era and, in countries without rotavirus vaccination programs in recent years [12–15]; thus, changes in clinical epidemiology might be accompanied by changes in molecular epidemiology. In previous studies, this association was often observed when the distribution of major circulating genotypes underwent changes [13].

Determination of circulating rotavirus genotypes is based on characterization of the capsid-protein-coding genes VP7 (G genotype) and VP4 (P genotype). To date, there are 27 G and 37 P genotypes have been identified (http://rotac.regatools.be/classificationinfo.html). Among them, five G genotypes (G1-G4, and G9) and three P genotypes (P[4], P[6], and P[8]) are most frequently detected. Surveillance by the World Health Organization (WHO) reported that five combined G/P genotypes (G1P[8], G2P[4], G3P[8], G4P[8], and G9P[8]) were considered globally prevalent during 2009-2012 [1]. However, there is regional and temporal variation in the most prevalent genotypes. Studies have shown that 70–90 % of all typeable genotypes in Korea belong to one of the five most common combined G/P genotypes or G4P[6] [16–20]. Between the years 1989 and 2009, the most prevalent genotype combination was G1P[8], though there were temporal changes in genotype predominance. For example, G1P[8] was predominant during 1987–2000 [21] and again between 2004 and 2009 [20, 22, 23]. G2P[4] and G4P[6], which occurred mainly in neonates, were reported to be the most common strains during the intervening period, 2000–2003 [24]. G9 was first detected in 2002 and was identified in consecutive seasons mostly as G9P[8] [22, 23, 25], becoming the fifth most common genotype in Korea [17, 26]. After the introduction of rotavirus vaccines, G1P[8] has remained predominant, however, the prevalence of G2P[4] seemed to have increased around 2013 in Korea [27, 28]. especially in Seoul, compared to the early vaccination period, although only a few studies on the surveillance of genotypes are available in the literature [27–29].

Therefore, this study explored changes in the clinical epidemiology and genotype distribution among rotavirus-infected children from a single hospital in the southwestern area of Seoul between 2011 and 2014, a period corresponding to 4–7 years after the introduction of rotavirus vaccination. We also examined the correlations between genotypes and clinical factors including age, clinical severity and vaccination status.

## Methods

### Patients and clinical information

This study was conducted prospectively in the pediatric ward of Seoul Metropolitan Government-Seoul National University Boramae Medical Center in Korea with the approval of the Seoul Metropolitan Government-Seoul National University Boramae Medical Center Institutional Review Board (IRB No.02-2011-11, 26-2014-142). Between November 2010 to October 2014, stool samples were collected from children hospitalized with a clinical suspicion of acute gastroenteritis (AGE) without known underlying chronic illnesses with immunosuppression within three days of admission. The patients were admitted via either the emergency department or outpatient clinics. Informed written consent was obtained from the parents or guardians of each child at enrollment. Patients were excluded based on the following criteria: failure to obtain permission, insufficient fecal samples, children with newly diagnosed chronic illnesses including malnutrition, those with a final co-diagnosis as other acute febrile illnesses including pneumonia, or suspected cases of secondary infections (nosocomial gastroenteritis) during their hospital stay.

Clinical information including age of onset, date of hospitalization, fever, duration and maximum number of diarrhea episodes, and duration and maximum number of vomiting episodes before and during hospitalization and at previous visits before admission (outpatient or emergency department) were collected. From these records, modified Vesikari scores [11] were assessed. Laboratory tests for electrolytes were performed for all children. For most children (>95 %), a stool test for white blood cells and *Salmonella* species (spp.), *Shigella* spp., and *Cholera* spp. were also performed by the hospital laboratory. For children who tested positive for rotavirus stool antigen, a vaccination history was obtained from the individual vaccination cards and the Korean Center for Disease Control website (http://is.cdc.go.kr), which records histories as part of the national vaccination program.

Additionally, to compare the results of clinical data from historical data before the introduction of vaccines in our hospital, electronic medical records from November 2003 to October 2015 were retrospectively reviewed and analyzed for the following information: age, date and year, diagnosis, and results of a rotavirus antigen test.

### Stool rotavirus test and genotyping

All stool samples were tested for rotavirus antigen by the hospital laboratory using the enzyme immunoassay kit Bioline rotavirus® (SD standard diagnostics, Youngin, Korea). Stool samples were diluted to 10 % with phosphate-buffered saline, centrifuged, and stored at −70 °C until further processing. For fecal specimens with positive stool antigen tests, viral dsRNA was extracted from stored samples using the QIAamp Viral RNA Mini Kit (Qiagen, Hilden, Germany). For genotyping, the extracted RNA was denatured and reverse transcription polymerase chain reaction (RT-PCR) was performed by using the Qiagen OneStep RT-PCR kit (Qiagene, Hilden, Germany). The VP7 gene was amplified by using primer sets Beg9 and End9 under conditions described previously [30, 31]. Second-round genotyping was analyzed using a pool of multiple primer sets aBT1, aCT2, aET3, aDT4, aAT8, aFT9 and G12, specific to G genotypes 1, 2, 3, 4, 8, 9 and 12, respectively [30–35]. To prevent known mistyping, additional semi-nested PCR for G3 and G9 typing was performed separately with different primers, G3 and G9 [36]. Second-round PCR for G5 and G10 was performed with the reverse primers FT5 and G10 [33]. The VP4 gene was amplified by using the primer sets Con3 and Con2 under conditions described previously [37]. P genotypes were analyzed by semi-nested PCR using a pool of multiple primer sets 2T1, 3T1, 1T1, 4T1, 5T1, and P11, specific to P genotypes 4, 6, 8, 9, 10, and 11, respectively [32, 37]. For P[4], P[6] and P[8] genotyping, additional semi-nested PCR was performed separately with a mentioned primer sets. For strains that were not P-typeable by genotyping PCR as mentioned above, the primer sets VP4F and VP4R were used to amplify for the VP4 gene under previously described conditions [38]. Amplification products were examined by electrophoresis in a 2 % agarose gel and documented with the Bio-Rad Gel Doc 1000 Documentation System (BioRad, Hercules, CA, USA).

### Nucleotide sequencing

For strains that were not G- or P-typeable by genotyping PCR, semi-nested PCR was performed with VP7 primers or VP4 primers after first round PCR [32, 38]. The resulting second-round amplicons were purified using the QIAquick Gel Extraction Kit (Qiagen) and directly sequenced using ABI Prism BioDye terminator cycle version 3.1 and an automatic DNA sequencer ABI 3730 (Applied Biosystems, Foster City, CA, USA). In addition, to validate the genotyping, one fecal sample corresponding to each of the G genotypes (G1,G2,G3,G4, and G9) and P genotypes (P[4], P[6] and P[8]) were sequenced using first round PCR products or second-round amplicons. The second-round amplicons of four randomly chosen strains with multiple P genotyping were also sequenced to confirm the results. The resulting nucleotide sequences were analyzed with the Bioedit software package. Genotypes were determined using the NCBI BLAST nucleotide search program in the GenBank database. The sequences of P untypeable strains were submitted to GeneBank under the accession numbers KR611086-KR611099.

### Statistical analysis

We examined the data for normality by using the Kolmogorov-Smirnov test for normality. For age-related analyses, both the monthly age and age groups (four groups: <3 months, 3–23 months, 24–59 months, and ≥60 months) were used. For comparison among years, four seasons were defined like the following as the rotavirus epidemic starts around November: 2011 season (November 2010 to October 2011), 2012 season (November 2011 to October 2012), 2013 season (November 2012 to October 2013), and 2014 season (November 2013 to October 2014). For comparison among periods from retrospective data, three periods were defined like the following: pre-vaccine period (November 2003 to October 2006), early vaccine period (November 2007 to October 2010), and the post-vaccine period (November 2011 to October 2015). Comparisons of categorical data were evaluated using the Chi-square test. For age groups and clinical severity-related categorical data, P value for Linear-by-linear test were used. Continuous variables were summarized using the median and interquartile range (IQR). Comparisons of continuous data were evaluated using the Mann–Whitney test or Kruskal-Wallis test. For the adjustment of genotype-associated factors, data were analyzed using multiple logistic regression with genotypes as the dependent variables and season, monthly age, and vaccination status as explanatory variables. The analysis using ordered logit models was also performed to determine the cumulative odds ratio for clinical severity-related factors as dependent variables and monthly age, vaccination status, and genotype as explanatory variables. Ordinal logistic regression analysis was performed using SAS Version 9.2 (SAS Institute, Cary, NC, USA). All other analyses were performed using SPSS Version 20.0 (IBM, Chicago, IL, USA).

## Results

### Overall prevalence, seasonal variation, and distribution of age

From 820 samples meeting the inclusion criteria during the study period, 179 samples (21.8 %) positive for rotavirus antigen were genotyped. The results of tests for other pathogen performed in the hospital laboratory were all negative for 179 samples.

**Fig. 1:**
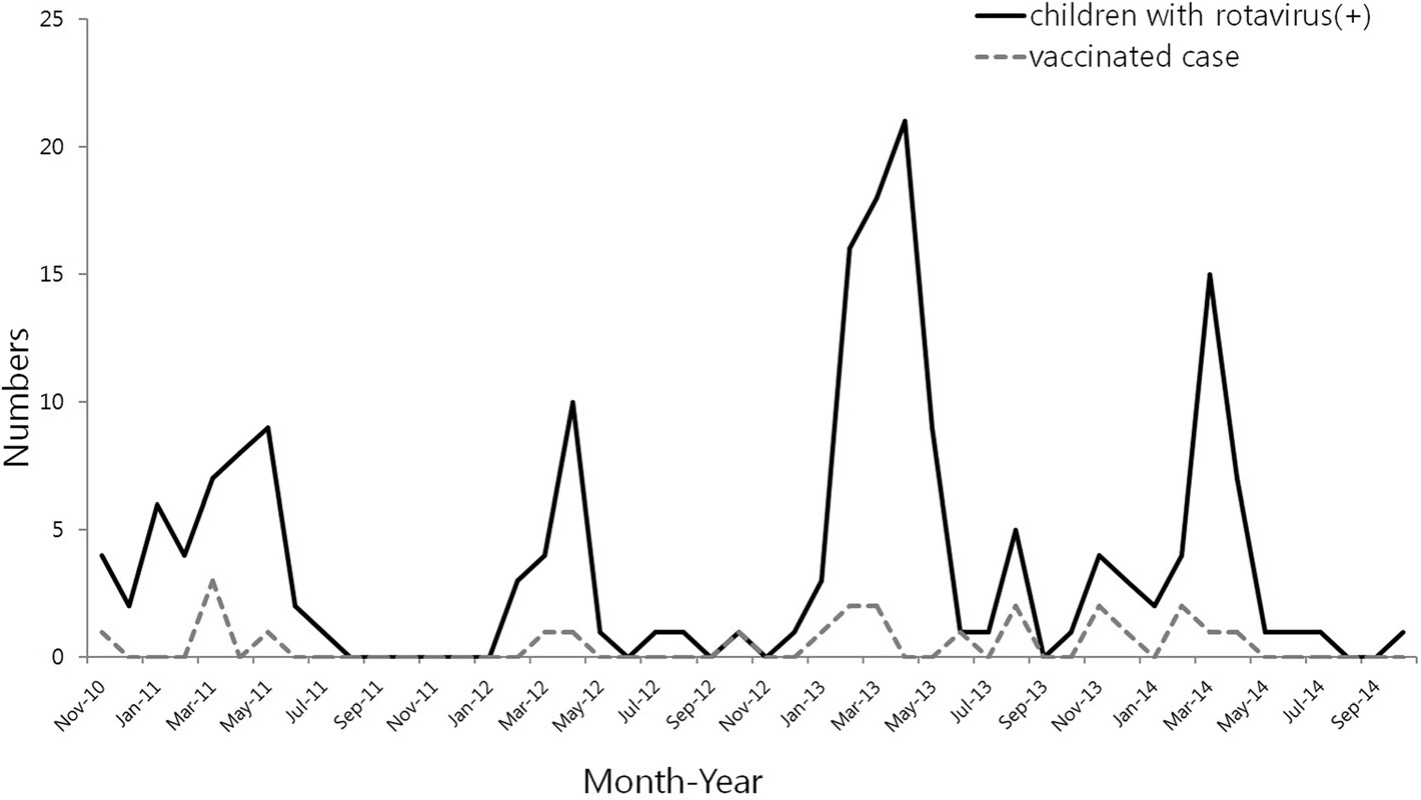
Seasonal distribution of rotavirus infection in children (*n* = 179)

From the retrospective data, the prevalence of rotavirus infection (352/2185 = 15.8 %, from November 2011 to October 2015) was decreased significantly compared to that of the pre-vaccine period (300/1152 = 25.9 %, from November 2003 to October 2006) and early-vaccine period (261/1367 = 18.9 %, from November 2007 to October 2010) (*P* = 0.000). Rotavirus infection was prevalent between January and May, peaking in March without significant variation among the four study seasons (Fig. 1). However, a delay in the peak season during the 2011–2014 seasons compared to the pre-vaccine period was observed (Additional file 1: Figure S1). The median age of the 179 children was 30.0 months [IQR 17.0–50.0], which was similar to the median age of the 312 children (28.6 months [IQR 14.0–48.0]) from the retrospective data during the 2011–2014 seasons. Of 179 children, 144 (80.4 %) were under five years of age. The rotavirus infection was more prevalent in children older than 24 months of age (*n* = 108) than those 3–23 months of age (*n* = 59) (Table 1).

**Table 1:** Clinical characteristics of 179 children with rotavirus infection including age, vaccination status and clinical severity

Clinical Parameters	Total	<3 month	3–23 months	24–59 months	≥60 months	*P* value^*^
	(*n* = 179)	(*n* = 12)	(*n* = 59)	(*n* = 73)	(*n* = 35)	
Ages, months	30 [17.0–50.0]	0.85 [0.6–2.0]	16.5 [12.0–21.5]	37.5 [29.3–45.00]	80.5 [71.5–100.5]	
Vaccination status, *n* (%)						
complete	19 (10.6)	0	10	9	0	0.09
rotateq	14	0	10	4	0	0.007
rotarix	5	0	0	5	0	0.179
incomplete	6	4	1	1	0	
Modified Vesikari score	12 [11–13]	9 [8.25–12]	12 [11–13]	12 [11–13]	12 [10–13]	0.565
≥11, *n* (%)	135 (75.4)	4 (33.3)	47 (79.7)	58 (79.5)	26 (74.3)	0.777
Vomiting duration, h, *n* (%)						0.007
0	21 (11.7)	7	8	5	1	
1–24	137 (76.5)	4	50	57	26	
25–48	17 (9.5)	1	0	9	7	
≥49	4 (2.2)	0	1	2	1	
Maximum number of episodes per 24-h, *n* (%)						0.036
0	21 (11.7)	7	8	5	1	
1	21 (12.1)	1	7	9	4	
2–4	81 (45.3)	3	30	33	15	
≥5	56 (31.3)	1	14	26	15	
Diarrhea duration, h, n (%)						
0	24 (13.4)	1	4	15	4	0.042
1–96	148 (82.7)	11	51	55	31	
97–120	3 (1.7)	0	2	1	0	
≥121	4 (2.2)	0	2	2	0	
Maximum number of episodes per 24-h, *n* (%),						0.021
0	24 (13.4)	1	4	15	4	
1–3	57 (31.8)	4	16	22	15	
4–5	39 (21.8)	4	15	12	8	
≥6	59 (33.0)	3	24	24	8	
Fever, °C, n (%)						0.791
≤37.0	21 (11.7)	3	4	10	4	
37.1–38.4	76 (42.5)	6	27	28	15	
38.5–38.9	49 (27.4)	1	18	20	10	
≥39.0	33 (18.4)	2	10	15	6	
Visit to ER, n (%)	105 (58.7)	7	34	49	15	0.838
Seizure, n (%)	6 (3.4)	1	2	3	0	0.614
Total CO2, mmol/L	17 [15.0–19.0]	20.5 [17.0–23.5]	16 [14.0–18.0]	17 [15.0–9.0]	18 [16.0–21.0]	0.031

*children between 3 and 23 month compared to those more than 24 months

**Fig. 2:**
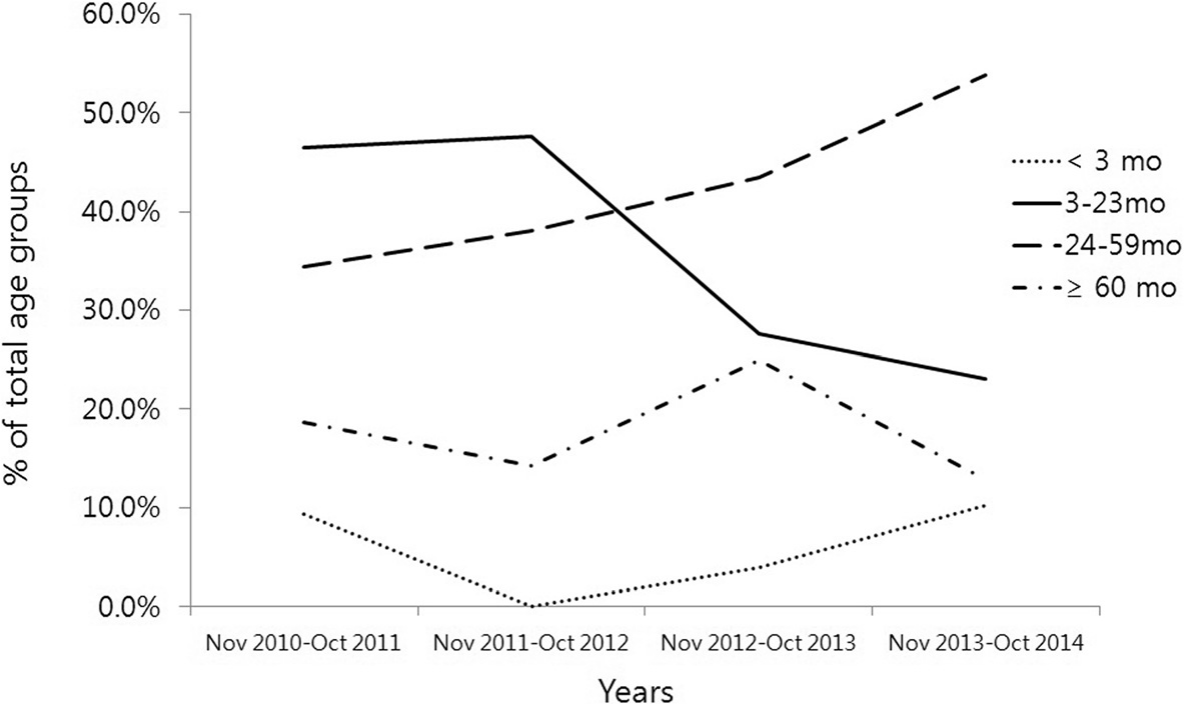
Age distribution of rotavirus infection during 2011–2014 seasons (*n* = 179). All age groups, *P* = 0.073;*, between 3–23 months and more than 24 months age groups, *P* = 0.010

The age of children infected with rotavirus was not significantly different between seasons during the study periods (*P* = 0.073). However, the number of children between three and 23 months of age decreased significantly over time compared to the number of children older than 24 months of age (*P* = 0.010) (Fig. 2). In addition, the median age of children with rotavirus infection significantly increased compared to the pre-vaccine or early vaccine periods (Additional file 2: Figure S2). The prevalence of the rotavirus infection compared to that of overall acute gastroenteritis was decreased mostly in children between three and 23 months of age, but was rather increased in children under 3 months of age and those over 60 months of age (Additional file 3: Figure S3).

### Clinical manifestations by age

Clinical severity based on a modified Vesikari score is summarized in Table 1. According to age, the neonate group had a lower modified Vesikari score than older age groups (*P* = 0.001). They also had a shorter duration of vomiting (*P* < 0.001), fewer vomit episodes (*P* < 0.001), and shorter duration of diarrhea (*P* = 0.046) than older age groups. After excluding the neonate group (*n* = 167), children more than two years of age had a significantly longer duration of vomiting (*P* = 0.007) and a more vomiting episodes (*P* = 0.036) than children younger than two years of age.

For diarrhea-related severity, children less than two years of age, excluding neonates, had a significantly longer duration of diarrhea (*P* = 0.042) and more diarrheal episodes (*P* = 0.021) than children older than two years. There were no differences in other clinical severity-related factors between the groups.

### Clinical manifestations by vaccination status

Excluding six children with incomplete vaccination records, 173 samples were analyzed for vaccination status. Of these children, 19 had completed rotavirus vaccination, 14 with RotaTeq and 5 with Rotarix (Table 1). The proportion of vaccinated children did not change over time and was significantly higher in children under five years of age (17.1 %, 19/111) than in children over 5 years (0 %). The median age of completely vaccinated children was 23.0 months [IQR 18.0–29.0], and the median age of unvaccinated children was 32.0 months [IQR 18.0–55.5] (*P* = 0.025).

As neonatal age significantly affected clinical severity-related factors, we analyzed data after excluding children under three months of age (*n* = 165). Vaccinated children had shorter durations of vomiting (*P* = 0.045) and fewer vomiting episodes (*P* = 0.012) (Table 2). There was no difference in diarrhea-related severity or other clinical severity including the overall Vesikari scores with vaccination status.

**Table 2:** Clinical characteristics of 165 children with rotavirus infection according to vaccination status excluding neonates and those incompletely vaccinated

Clinical Parameters	Total	Vaccinated	Unvaccinated	*P* value
	(*n* = 165)	(*n* = 19)	(*n* = 146)	
Ages, months	30.5 [19.8–53.8]	23 [18–29]	34.5 [20.8–57.8]	0.007
Modified Vesikari score	12 [11–13]	12 [11–13]	12 [11–13]	0.887
≥11, *n* (%)	129 (78.2)	16 (9.7)	113 (68.5)	0.500
Vomiting duration, h, *n* (%)				0.045
0	13 (7.9)	4	9	
1–24	132 (80.0)	14	118	
25–48	16 (9.7)	1	15	
≥49	4 (2.4)	0	4	
Maximum number of episodes per 24-h, n (%)				0.012
0	13 (7.9)	4	9	
1	20 (12.1)	2	18	
2–4	77 (46.7)	11	66	
≥5	55 (33.3)	2	53	
Diarrhea duration, h, n (%)				0.852
0	23 (13.9)	1	22	
1–96	135 (81.8)	18	117	
97–120	3 (1.8)	0	3	
≥121	4 (2.4)	0	4	
Maximum number of episodes per 24-h, *n* (%),				0.175
0	23 (13.9)	1	22	
1–3	52 (31.5)	5	47	
4–5	35 (21.2)	5	30	
≥6	55 (33.3)	8	47	
Fever, °C, *n* (%)				0.219
≤37.0	18 (10.9)	0	18	
37.1–38.4	70 (42.4)	8	62	
38.5–38.9	46 (27.9)	7	39	
≥39.0	31 (18.8)	4	27	
Visit to emergency room, n (%)	95 (57.6)	11	84	0.976
Seizure, n (%)	5 (3.0)	1	4	0.783
Total CO2, mmol/L	17 [14–19]	17 [14–20]	17 [14.8–19]	0.904

Because vomiting-related severity was also affected by age, the analysis using ordered logit models was performed. Vomiting duration was significantly associated with monthly age (cumulative odds ratio, c-OR, 1.017; 95 % CI, 1.005–1.029; *P* = 0.006). However, there was a significant association between the maximum number of vomiting episodes per 24 h and vaccination status (c-OR, 0.339; 95 % CI, 0.138-0.832; *P* = 0.018).

### Overall prevalence and seasonal variation of rotavirus genotypes

The G genotype was identified in 97.2 % (*n* = 174) and P genotype in 97.8 % (*n* = 175) of samples. G1 was the most common G genotype (48.0 %, *n* = 86), followed by G2 (24.6 %, *n* = 44), G3 (20.1 %, *n* = 36), G9 (10.6 %, *n* = 19), and G4 (3.4 %, *n* = 6). P[4] was the most prevalent P genotype (68.2 %, *n* = 122). P[8] was found in 45.3 % (*n* = 81), and P[6] in 3.9 % (*n* = 7). Co-infections with P[4] and P[8] genotypes were identified in 19.6 % of cases (*n* = 35). In G/P combinations, G2P[4] was the most prevalent, found in 18.4 % (*n* = 33) of cases, followed by G1P[8] (14.5 %, *n* = 26) and G1P[4]P[8] (13.4 %, *n* = 24), although the most common genotypes were different among seasons (Table 3).

**Table 3:** Distribution of group A rotavirus G/P genotypes during the fourth to seventh year after the introduction of vaccines, n (%)

	Nov 2010–Oct-11	Nov 2011–Oct-12	Nov 2012–Oct-13	Nov 2013–Oct-14	Total
Common genotypes					
G2P[4]	8 (18.6)	1 (4.8)	17 (22.4)	7 (17.9)	33 (18.4)
G1P[8]	5 (11.6)	8 (38.1)	12 (15.8)	1 (2.6)	26 (14.5)
G3P[8]	3 (7.0)	1 (4.8)	3 (3.9)	2 (5.1)	9 (5.0)
G9P[8]	1 (2.3)	2 (9.5)	1 (1.3)	1 (2.6)	5 (2.8)
G4P[6]	0	0	0	1 (2.6)	1 (0.6)
G4P[8]	1 (2.3)	0	0	0	1 (0.6)
subtotal	18 (41.9)	12 (57.1)	33 (43.4)	12 (30.8)	75 (41.9)
Uncommon genotypes					
G3P[4]	3 (7.0)	4 (19.0)	10 (13.2)	2 (5.1)	19 (10.6)
G1P[4]	4 (9.3)	0	4 (5.3)	10 (25.6)	18 (10.1)
G9P[4]	2 (4.7)	1 (4.8)	2 (2.6)	2 (5.1)	7 (3.9)
G1P[6]	0	0	1 (1.3)	1 (2.6)	2 (1.1)
G2P[8]	0	0	1 (1.3)	0	1 (0.6)
subtotal	9 (20.1)	5 (23.8)	18 (23.7)	15 (38.4)	47 (26.3)
Multiple genotypes					
G1P[4]P[8]	8 (18.6)	0	9 (11.8)	7 (17.9)	24 (13.4)
G1G2P[4]	2 (4.7)	1 (4.8)	3 (3.9)	1 (2.6)	7 (3.9)
G9P[4]P[8]	0	2 (9.5)	0	2 (5.1)	4 (2.2)
G3P[4]P[8]	0	0	3 (3.9)	1 (2.6)	4 (2.2)
G1G4P[6]	1 (2.3)	0	1 (1.3)	1 (2.6)	3 (1.7)
G1G3P[4]P[8]	1 (2.3)	0	1 (1.3)	0	2 (1.1)
G1G9P[8]	0	0	2 (2.6)	0	2 (1.1)
G1G3P[8]	0	0	1 (1.3)	0	1 (0.6)
G1G4P[8]	1 (2.3)	0	0	0	1 (0.6)
G2G3P[4]	1 (2.3)	0	0	0	1 (0.6)
G2P[4]P[8]	1 (2.3)	1 (4.8)	0	0	2 (1.1)
subtotal	15 (34.9)	4 (19.0)	20 (26.3)	12 (30.8)	51 (28.5)
nontypeable					
none	1 (2.3)	0	2 (2.6)	0	3 (1.7)
P[4]	0	0	2 (2.6)	0	2 (1.1)
G9	0	0	1 (1.3)	0	1 (0.6)
subtotal	1 (2.3)	0	5 (6.5)	0	6 (3.4)
Total	43 (100)	21 (100)	76 (100)	39 (100)	179 (100)

Overall, the five most common WHO combinations were identified in 41.3 % of cases (*n* = 74). Conversely, genotypes with unusual P[4] combinations including G1P[4], G3P[4] and G9P[4] were prevalent in 24.6 % of cases (*n* = 44). Interestingly, G3P[4] and G9P[4] were more prevalent than G3P[8] and G9P[8]. Co-infection with mixed G/P genotypes was detected in 28.5 % of cases (*n* = 51). The proportion of uncommon genotypes including mixed type was more than 40-50 %, which was consistent among the four seasons (Table 3). There were no significant differences in the frequencies of other single or multiple G, P, or G/P combinations between seasons.

### Rotavirus genotypes by age

There was no age difference between common, uncommon and/or mixed genotypes. However some G, P or G/P genotypes showed an age preference (Table 4).

**Table 4:** Distribution of group A rotavirus G/P genotypes by age groups, n (%)

	<3 months	3–23 months	24–59 months	≥60 months	Total	*P* value*
Common genotypes						
G2P[4]	0	7 (21.2)	16 (48.5)	10 (30.3)	33 (18.4)	0.002
G1P[8]	2 (7.7)	12 (46.2)	10 (38.5)	2 (7.7)	26 (14.5)	0.050
G3P[8]	0	5 (55.6)	3 (33.3)	1 (11.1)	9 (5.0)	0.506
G9P[8]	0	1 (20.0)	2 (40.0)	2 (40.0)	5 (2.8)	0.126
G4P[6]	1	0	0	0	1 (0.6)	
G4P[8]	0	0	1	0	1 (0.6)	
subtotal	3	25	32	15	75 (41.9)	0.377
Uncommon genotypes						
G3P[4]	0	7 (36.8)	12 (63.2)	0	19 (10.6)	0.992
G1P[4]	3 (16.7)	6 (33.3)	6 (33.3)	3 (16.7)	18 (10.1)	0.329
G9P[4]	0	3 (42.9)	2 (28.6)	2 (28.6)	7 (3.9)	0.400
G1P[6]	1	1	0	0	2 (1.1)	0.023
G2P[8]	0	0	1	0	1 (0.6)	
subtotal	4	17	21	5	47 (26.8)	0.235
Multiple genotypes						
G1P[4]P[8]	2 (8.3)	3 (12.5)	9 (37.5)	10 (41.7)	24 (13.4)	0.010
G1G2P[4]	0	4 (57.1)	2 (28.6)	1 (14.3)	7 (3.9)	0.104
G9P[4]P[8]	0	1 (25)	3 (75)	0	4 (2.2)	
G3P[4]P[8]	0	2 (50)	2 (50)	0	4 (2.2)	
G1G4P[6]	3 (100)	0	0	0	3 (1.7)	0.006
G1G3P[4]P[8]	0	2	0	0	2 (1.1)	
G1G9P[8]	0	1	1	0	2 (1.1)	
G1G3P[8]	0	1	0	0	1 (0.6)	
G1G4P[8]	0	1	0	0	1 (0.6)	
G2G3P[4]	0	0	0	1	1 (0.6)	
G2P[4]P[8]	0	2	0	0	2 (1.1)	
subtotal	5	17	17	12	51 (28.5)	0.707
nontypeable						
none	0	0	2	1	3 (1.7)	
P[4]	0	0	0	2	2 (1.1)	
G9	0	0	1	0	1 (0.6)	
Subtotal	0	0	3	3	6 (3.4)	0.025
Total	12	59	73	35	179 (100)	

*compared to other genotypes

Children infected with G1 (median, 22.5 months; [IQR 12.5–34.5]) were younger than children with the G2 (median, 41.0; [IQR 25.0–61.9]; *P* = 0.001), and G9 (median, 39.0 months; [IQR 22.5–56.3]; *P* = 0.010) genotypes after exclusion of G and P type co-infection cases. Children infected with P[4] (median, 31.5 months [IQR 21.5–53.8]) were older than children with P[8] (median, 26.0 months [IQR 15.3–42.1; *P* = 0.049) after exclusion of G and P type co-infection cases. However, the age preference of the aforementioned genotypes was not significant after adjustment for seasons in logistic regression analysis.

Among G/P combinations, the most predominant genotypes were G1P[4] and G1G4P[6] in neonates (both 27.3 %, *n* = 3), G1P[8] at 3–23 months (20.0 %, *n* = 12), G2P[4] at 24–59 months (21.9 %, *n* = 16), and G2P[4] and G1P[4]P[8] at ≥60 months (both 28.6 %, *n* = 10). The prevalence of G2P[4], G1P[4]P[8], and untypeable type was significantly higher in older age groups (*P* = 0.002, 0.010, and 0.025 respectively). G1G4P[6] was identified only in neonates (*P* < 0.001) (Table 2). The age of children infected with G2P[4] (median, 41.5 months [IQR 25.0–62.75]) or G1P[4]P[8] (median, 44.5 months [IQR 28.5–78.5]) was significantly older than G1P[8]-infected children (median, 22.25 months [IQR 13.88–30.63]; *P* = 0.001 and *P* = 0.005, respectively). Compared to G1P[8], this age preference for older children was still significant after adjusting for seasons and vaccination status in the G2P[4] (adjusted odds ratio, a-OR of monthly age, 1.042; 95 % confidence interval, CI, 1.010–1.075; *P* = 0.011) and G1P[4]P[8] (a-OR, 1.043; 95 % CI, 1.009–1.077; *P* = 0.012) genotypes.

### Rotavirus genotype by vaccination status

There were no significant differences between the two groups according to the vaccination status in the frequencies of common, uncommon, mixed genotypes, or combinations of G/P genotypes according to vaccination status (Additional file 4: Table S1).

However, among single G/P genotypes, G9 genotype was detected more frequently in the vaccinated group than in the unvaccinated group (*n* = 5/19; 27.8 % vs *n* = 14/154; 9.3 %, respectively; *P* = 0.020) (Additional file 5: Table S2). The association with G9 and vaccination status was still significant (a-OR of vaccination, 5.261; 95 % CI, 1.477–18.734; *P* = 0.010) after adjustment for seasons and age (a-OR of monthly age, 1.014; 95 % CI, 0.9996–1.028; *P* = 0.036) in multiple logistic regression.

### Rotavirus genotypes and clinical severity

As neonatal age significantly affected clinical severity-related factors, we analyzed data after excluding children under three months of age (*n* = 167). There was no significant difference in clinical severity among common, uncommon and mixed genotypes. However, some clinical severity was significantly different among some G, P or G/P genotypes (Table 5).

**Table 5:** Clinical characteristics of G1P[8], G2P[4], and G1P[4]P[8] in 167 children more than 3 months of age

Clinical		G1P[8]	G2P[4]	G1P[4]P[8]	*P*value*	*P* value**	*P* value***
		(*n* = 24)	(*n* = 33)	(*n* = 22)			
Ages, median months		24.0 [14.5–31.5]	42.0 [25.0–62.5	45.0 [29.5–79.0]	0.005	0.003	0.122
Complete vaccination, *n* (%)		2 (8.3)	3 (9.1)	1 (4.5)	0.921	0.344	0.529
Modified Vesikari score, median		11 [10–12]	12 [11–13]	12 [10–14]	0.077	0.230	0.889
Modified Vesikari score ≥ 11, *n* (%)		17 (70.8)	27 (81.8)	16 (72.7)	0.333	0.888	0.428
Vomiting duration, h, *n* (%)	0	5 (20.8)	0	2 (9.1)	0.018	0.165	0.535
	1–24	17 (70.8)	27 (81.8)	16 (72.7)			
	25–48	2 (8.3)	5 (15.2)	3 (13.6)			
	≥ 49	0	1 (3.0)	1 (4.5)			
Maximum number of episodes per 24-h, *n* (%)	0	5 (20.8)	0	2 (9.1)	0.006	0.091	0.428
	1	5 (20.8)	3 (9.1)	2 (9.1)			
	2–4	9 (37.5)	16 (48.5)	10 (45.5)			
	≥ 5	5 (20.8)	14 (42.4)	8 (36.4)			
Diarrhea duration, h, *n* (%)	0	2 (8.3)	6 (18.2)	3 (13.6)	0.180	0.973	0.283
	1–96	21 (87.5)	27 (81.8)	17 (77.3)			
	97–120	1 (4.2)	0	1 (4.5)			
	≥ 121	0	0	1 (4.5)			
Maximum number of episodes per 24-h, *n* (%),	0	2 (8.3)	6 (18.2)	3 (13.6)	0.161	0.454	0.571
	1–3	7 (29.2)	13 (39.4)	8 (36.4)			
	4–5	6 (25.0)	5 (15.2)	4 (18.2)			
	≥ 6	9 (37.5)	9 (27.3)	7 (31.8)			
Fever, °C, *n* (%)	≤ 37.0	4 (16.7 %)	3 (9.1 %)	1 (4.5 %)	0.229	0.220	0.978
	37.1–38.4	12 (50 %)	14 (42.4 %)	10 (45.5 %)			
	38.5–38.9	4 (16.7 %)	8 (24.2 %)	7 (31.8 %)			
	≥ 39.0	4 (16.7 %)	8 (24.2 %)	4 (18.2 %)			
Visit to emergency department, *n* (%)		15 (62.5)	17 (51.5)	10 (45.5)	0.413	0.252	0.663
Seizure, *n* (%)		1 (4.2)	1 (3.0)	0	0.156	0.338	0.414
Total CO2, median mmol/L		16.0 [14.0–18.0]	17.0 [14.5–18.5]	17.5 [14.8–19.0]	0.769	0.244	0.337

*G2P[4] compared to G1P[8]

**G1P[4]P[8] compared to G1P[8]

***G2P[4] compared to G1P[4]P[8]

Children (*n* = 167) infected with the G2 genotype presented with longer durations of vomiting (*P* = 0.030) and more vomiting episodes (*P* = 0.007) than those infected with G1, after exclusion of G/P co-infection cases. Children infected with the P[8] genotype had longer durations of diarrhea (*P* = 0.025), but not more diarrheal episodes (*P* = 0.070) than those infected with the P[4] genotype, after exclusion of G/P co-infection cases. There were no differences in clinical severity among other G or P genotypes.

For G/P combinations, we compared G1P[8] and other major G/P combinations, including G2P[4]. Children with G2P[4] were older (*P* = 0.005) and had longer durations of vomiting (*P* = 0.018), and more vomiting episodes (*P* = 0.006) than children with G1P[8]. Modified Vesikari scores did not show any significant differences between two genotypes (*P* = 0.077). Children with G1P[4]P[8] were older than those with G1P[8] (*P* = 0.003). However, there were no differences in the clinical manifestations of G1P[4]P[8] and G1P[8] (Table 5). Neither were there any significant differences in clinical manifestations between G1P[8] and other G/P combinations, including G1P[4], G3P[4], and G9P[4].

As older age, unvaccinated status, and infection with the G2P[4] genotype compared to G1P[8] were all significantly associated with greater vomiting-related clinical severity, the analysis using ordered logit models was performed. Genotype was found to be a significant factor for both vomiting duration (c-OR of G2P4 genotype after adjustment for age, 4.89; 95 % CI, 1.242–19.258; *P* = 0.023) and the maximum frequency of vomiting (c-OR of G2P4 genotype after adjustment for vaccination status, 4.68; 95 % CI, 1.658–13.232; *P* = 0.004); monthly age (c-OR for vomiting duration, 1.015; 95 % CI, 1.001–1.028; *P* = 0.031) and vaccination status (c-OR for maximum frequency of vomiting, 0.184; 95 % CI, 0.066–0.514; *P*-0.001) also remained as significant factors for vomiting-related severity after adjustment for genotypes.

## Discussion

This study spanned the fourth to seventh years of surveillance of rotaviruses in a single hospital in southwestern area of Seoul after the introduction of rotavirus vaccines in Korea. Our report contains two notable findings; firstly, it demonstrated a significant increase in the age of hospitalized children with the rotavirus infection after the introduction of the rotavirus vaccination program in Seoul, Korea. We further showed that this shift in infected age could be accompanied by a change in vomiting and diarrheal severity. Secondly, a distinctive change in the distribution of rotavirus genotypes, including a prominent increase in genotypes of uncommon and mixed combinations, was observed during the study periods. A relative increase of the G2P[4] genotype was also observed, which was significantly associated with age and clinical severity in this population. Although these are the results from a single hospital with a modest number of fecal samples, a considerable number of findings in clinical and molecular epidemiology share similar trends observed in other single center studies from Seoul and some multi-center Korean studies conducted in the post-vaccine period [2, 5, 10, 27–29]. To our knowledge, this is one of the first attempts to explore the link between age, clinical severity and genotypes in a region that adopted the rotavirus vaccination, which may contribute to an integrated understanding of the changing patterns in both clinical and molecular epidemiology for rotavirus gastroenteritis after the introduction of vaccines.

The finding of the shift of infected age tended to be more prominent the further in time it was separated from the year in which vaccines were introduced, which was consistent with the results of a few previous Korean studies [2, 10]. In a multicenter study, the prevalence of rotavirus infection compared to that of overall acute gastroenteritis was examined during 2007–2010. The ratio of children between less than 24 months old to more than 24 months old decreased from 1.2 (54.46 %/46.18 %) in 2007 to 0.7 (6.35 %/8.68 %) in 2010 [2]. In another, a single hospital study performed in the suburbs of Seoul, the median age of children with rotavirus infection increased to 3.6 years in 2012 from 1.7 years in 2007 [10]. Although less prominent, a similar shift in age from 14.9 months in the pre-vaccine period to 19 months in the post-vaccine period was also observed in a Greek study [39], in which the rotavirus vaccination rate was 25–30 %. The shift in infected age may partly reflect the protective effect of vaccines against severe rotavirus infection in vaccinated young children, who were the main focus of the newly introduced rotavirus vaccines in contrast to older children, who could not be vaccinated due to age restrictions or lack of information regarding the vaccines. However, the prevalence of the rotavirus infection, compared to that of overall acute gastroenteritis, actually increased significantly in children, especially those older than 60 months of age, compared to that of the pre-vaccine era, although it decreased significantly in the whole population (Additional file 3: Figure S3). We hypothesize that the changing distribution of the rotavirus genotypes, such as an increase of G2P[4] and G1P[4]P[8] that show older age preference in our study, may also be associated with this possibly transient increase in the age group of infected children, in addition to the age group deviation of vaccine recipients.

Interestingly, we found a significant association between clinical severity and age partly due to shift in age, which could produce a sufficient number of relatively old age children (including approximately 20 % of children older than 60 months) comparable to that of younger age children. Vomiting-related severity increased and diarrhea-associated severity decreased with age in rotavirus gastroenteritis. Although it is controversial if and how clinical severity is associated with age [40, 41], this result is compatible with our speculation, as pediatricians in clinical practice, that vomiting has become the main symptom of rotavirus gastroenteritis, with diarrheal severity becoming less prominent in the last few years. This may be associated with an increase in patient age after the introduction of vaccines. In our study, clinical severity was also related to vaccination status, although the difference was not very impressive, as well as different genotypes in addition to age. On the basis of the distribution of these factors in the study subjects and the clinical severity-related factors examined, we speculate that either older (vomiting-related severity) or younger (diarrhea-related severity) age could be associated with greater clinical severity, or, there could be no association between age and overall severity (as measured by the modified Vesikari score) [40, 41].

Regarding genotype distribution, two notable changes were observed during the study period. First, G2P[4] was the most prevalent genotype overall, though there was seasonal variation. The predominance of G2P[4], has been reported recently in many countries with or without vaccination including Korea [28, 42]. This change might be related to natural variation rather than vaccine pressure. Second, the prevalence of P[4] combinations with G1, G3 and G9 (G1P[4], G3P[4] and G9P[4]), along with their mixed P genotype combinations G1P[4]P[8], G3P[4]P[8], and G9P[4]P[8] increased to more than 40 % among typeable strains, although the G1, G3, and G9 genotypes were mostly found in combination with P[8] in the past [43]. As a result, the prevalence of the five most common WHO combinations was less than 50 %, which was relatively consistent during all four seasons. Until recently, most studies in Korea identified the five most common WHO combinations plus G4P[6] (mainly in neonates) in more than 70–90 % of typeable strains [16–20]. The genotypes of mixed G/P combinations were not prevalent in most studies. In a pre-vaccine era (2000–2007) study in Korea, 85.3 % were 5 common genotypes plus G4P[6], and uncommon genotypes and mixed genotypes were 11.8 % (5.82 % and 5.97 %, respectively) [44]. In other meta-analysis during 1989–2009 in Korea, 81.8 % were common genotypes [26]. However, a recent single hospital study in the northeastern area of Seoul reported a high prevalence of genotypes with non-WHO combinations and mixed G/P combinations similar to ours, in which the most prevalent G/P genotype was G9P[4] [29]. It has been known that the occurrence of mixed infection by more than two strains with different genotype combinations could encourage genotype reassortment and the appearance of strains with uncommon genotype combinations [45, 46]. Although increases in naturally occurring reassortment have often been reported [45], the unusual increase in uncommon and mixed genotypes observed in our study might reflect one of the responses to the introduction of rotavirus vaccines. Increases in previously uncommon G/P combinations, especially those not included in the current vaccines, have also been observed in other countries [9, 47–49]: In a recent study in Brazil, an increase of G3P[6] was reported following the introduction of the G1P[8] attenuated vaccine [49]. In the United States, an increase of G12 and a relatively low vaccine efficacy against this genotype were reported [48]. Although it has not been determined whether vaccine-associated selection pressure may play a role in these observations [9], studies have reported that increases in uncommon G/P combinations tend to coincide with the implementation of a rotavirus vaccination program [47–49]. Future large scale studies for prolonged periods, both covering clinical and molecular epidemiology, are needed to document the significance of the increase of genotypes of uncommon and mixed combinations in our population.

In our study, the distinctive difference among the major distribution of genotypes was not observed between unvaccinated children or those with vaccine failure: the prevalence of genotypes of uncommon or mixed type combinations or major G/P combinations such as G2P[4] was not significantly different between the two groups. Although the proportion of G9 genotype was significantly higher in the vaccinated group, the significance of this observation could not be determined because of a relatively low significant P value (*P* = 0.010) in a small number of vaccinated children (*n* = 19). In previous two Korean studies, although the number of vaccinated children was also small, there was no significant difference in the genotype distribution between vaccinated children and those unvaccinated [27, 28].

We found a significant relationship between certain genotypes and clinical factors, including age and vomiting- or diarrhea-related severity. In addition to the well-known neonatal preference of G4P[6] [50], we found that G2P[4], G1P[4]P[8], and G9 strains preferentially infected older children, after adjustment for season and vaccination status. Among them, G2P[4] was significantly associated with greater vomiting-related severity compared to other genotypes, including G1P[8], beyond neonatal age. It is not clear why children infected with strains with G2P[4] showed increased vomiting severity compared to those infected with G1P[8]. However, the association between G2P[4] and age or clinical severity itself is mostly consistent with the results of several studies from the pre-vaccine era [12, 13, 51] and in countries without rotavirus vaccination [14, 15]. The literature suggests that the preference of G2P[4] or G2 to infect older children and cause greater clinical severity might be a consequence of a G2 epidemic after a prolonged period of nonprevalence [13]. Cross-protective immunity may be less effective against G2 than other common G types or G2 may possibly be more virulent [12, 52]. These speculations are supported by previous studies on the predominance of G2 in children with second episodes of rotavirus gastroenteritis [14] or adult outbreaks of rotavirus gastroenteritis [52].

In Korea, G2P[4] was prevalent before 2002, with its incidence decreasing until recently, and was again prevalent in our study period [24, 28]. Children at ages that are vulnerable to rotavirus gastroenteritis (except rotavirus-naïve children) may be more susceptible to G2P[4] stains than G1P[8], which was prevalent until recently. Due to rotavirus vaccination, which began in 2007, many younger children have protective immunity against most common and possibly uncommon genotypes. However, many older children, who usually did not receive rotavirus vaccination in the early vaccine period due to an age restrictions or a lack of information regarding the vaccine, may have insufficient protective immunity against G2P[4] strains, probably because of a lack of type-specific immunity and low cross-protective immunity against G2P[4] strains compared to other common genotypes, including G1P[8]. Therefore, infection with G2P[4] strains occur more often in older children than with G1P[8] and other common genotypes, influenced by both the G2P[4] epidemic and vaccines. This association between genotype and age could also be one of the explanations for the significantly older age of unvaccinated children compared to younger children with vaccine failure in addition to age group deviation of the vaccine recipients.

There were some limitations in our study. First, this is not a large-scale multicenter study. Only hospitalized children at a single hospital were included. With regard to genotype-related clinical severity, community-based comparisons may yield more prominent and accurate differences between genotypes [53]. Second, the number of vaccinated children was small, so we could not conclude the genotypic comparison between vaccinated and unvaccinated groups. On the other side, the low proportion of vaccinated children in this study might imply the protective effect of rotavirus vaccine. Third, tests for co-infections with microbial agents other than *Salmonella* spp*.*, *Shigella* spp*.,* and *Cholera* spp. were not performed in our patients. However, a recent study reported that clinical severity was not affected by multiple co-infections with other enteric viruses [42], with rotavirus showing the most severe clinical manifestations [40]. Finally, other possible risk factors for severity such as previous breastmilk feeding or day care center attendance were not considered. However, children with major potential risk factors such as malnutrition or underlying chronic illnesses affecting host immunity were not included in our study.

## Conclusions

This prospective hospital-based study revealed that rotavirus vaccine showed fare efficacy in southwestern area of Seoul between 2011 and 2014, corresponding to the fourth to seventh years of rotavirus vaccination, although rotavirus vaccines were not included in the national vaccination program. Age of children with rotavirus gastroenteritis increased significantly after the introduction of vaccines among hospitalized children in Seoul, partly suggesting a protective effect of vaccines in recipients and a possible association between host age and virus genotypes. Clinical severity of rotavirus infection was associated with age, vaccination status, and certain genotypes in this population. Future large scale studies with long term surveillance of genotypes are needed to document the significance of the increase of genotypes of uncommon and mixed combinations.

## Abbreviations

a-OR, adjusted odds ratio; c-OR, cumulative odds ratio; IQR, interquartile range; RT-PCR, reverse transcription-polymerase chain reaction; spp, species

## Additional files

Additional file 1: Figure S1. Monthly distribution of rotavirus infections in children during pre-vaccine (2004–2006), early-vaccine (2008–2010), and post-vaccine (2011–2015) periods. (JPG 272 kb) Additional file 2: Figure S2. Age distribution of rotavirus infections in children during pre-vaccine (2004–2006), early-vaccine (2008–2010), and post-vaccine (2011–2015) periods. (JPG 668 kb) Additional file 3: Figure S3. Changes of the rotavirus infection rates by ages during pre-vaccine (2004–2006), early-vaccine (2008–2010), and post-vaccine (2011–2015) periods. (JPG 1221 kb) Additional file 4: Table S1. Distribution of group A rotavirus G and P genotypes according to vaccination status, *n* (%) (DOCX 45 kb) Additional file 5: Table S2. Distribution of group A rotavirus G and P genotypes according to the vaccination status (exclude incomplete vaccination cases, *n* = 173) (DOCX 34 kb)
