# Supplementary figures and images for: Changing distribution of age, clinical severity, and genotypes of rotavirus gastroenteritis in hospitalized children after the introduction of vaccination: a single center study in Seoul between 2011 and 2014
Source: BMC Infect Dis. 2016 Jun 14;16:287. doi: 10.1186/s12879-016-1623-y (PMC4906974; doi:10.1186/s12879-016-1623-y)

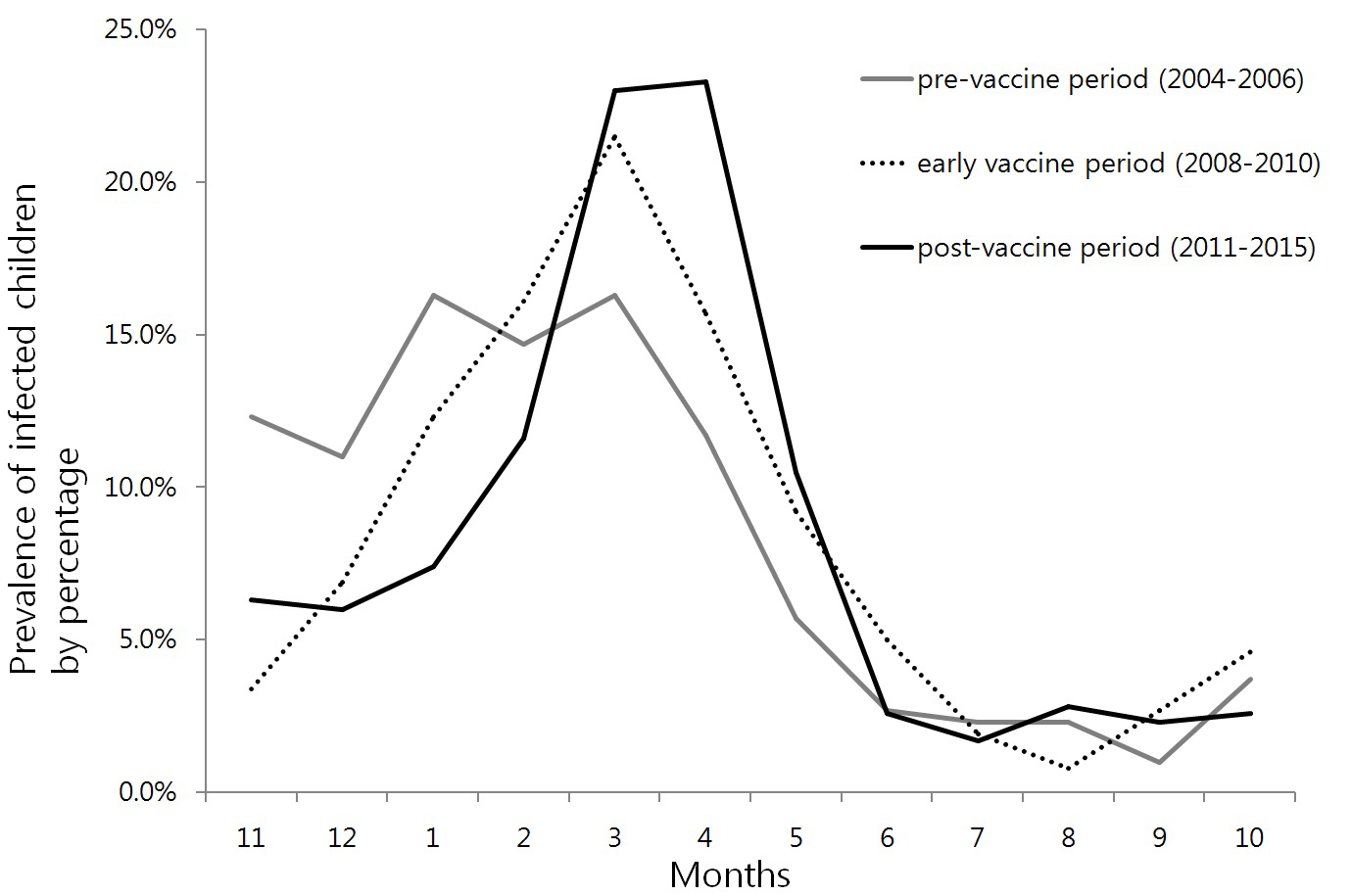

Supplement: Additional file 1: Figure S1. — Monthly distribution of rotavirus infections in children during pre-vaccine (2004–2006), early-vaccine (2008–2010), and post-vaccine (2011–2015) periods. (JPG 272 kb) [file 12879_2016_1623_MOESM1_ESM.jpg]

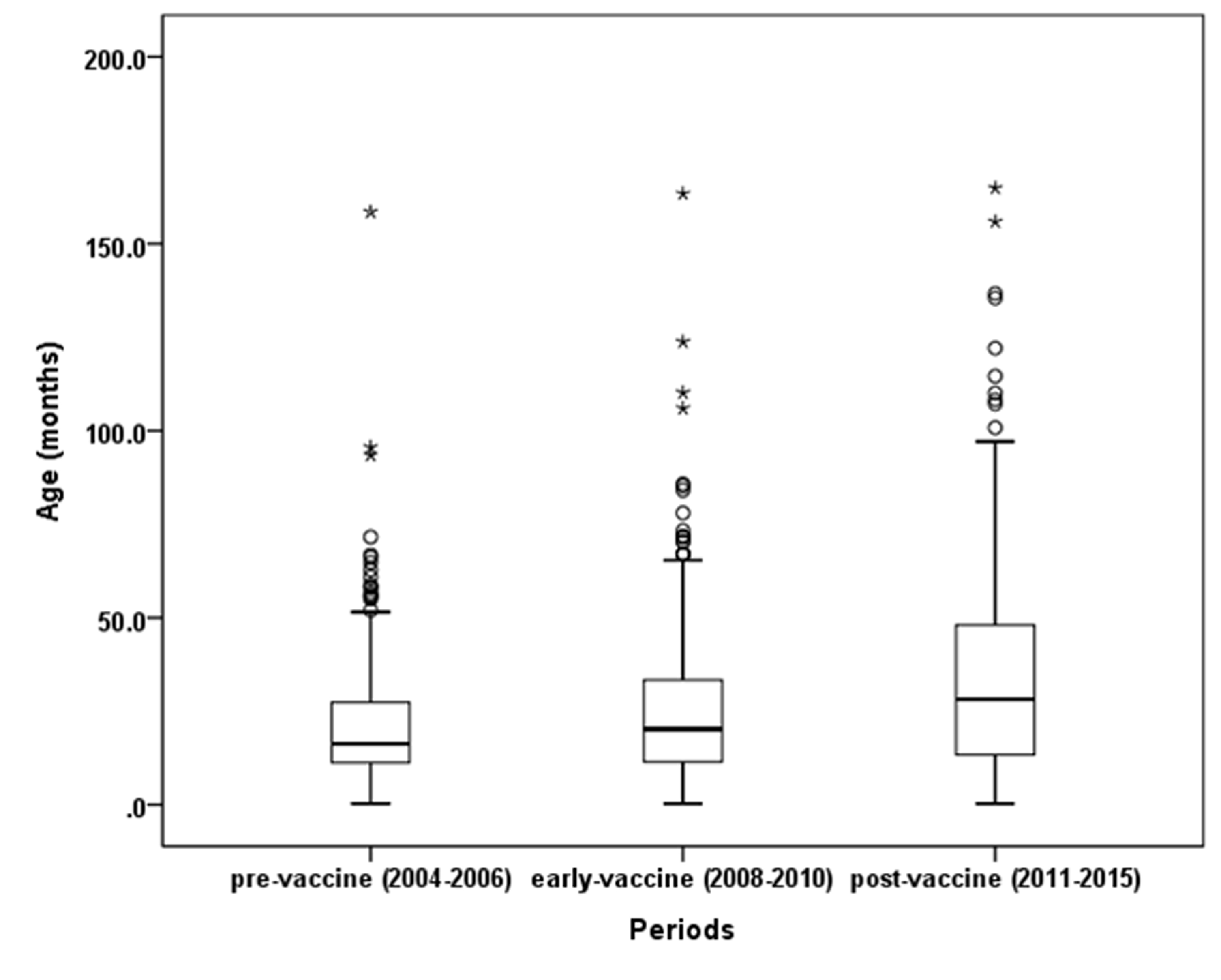

Supplement: Additional file 2: Figure S2. — Age distribution of rotavirus infections in children during pre-vaccine (2004–2006), early-vaccine (2008–2010), and post-vaccine (2011–2015) periods. (JPG 668 kb) [file 12879_2016_1623_MOESM2_ESM.jpg]

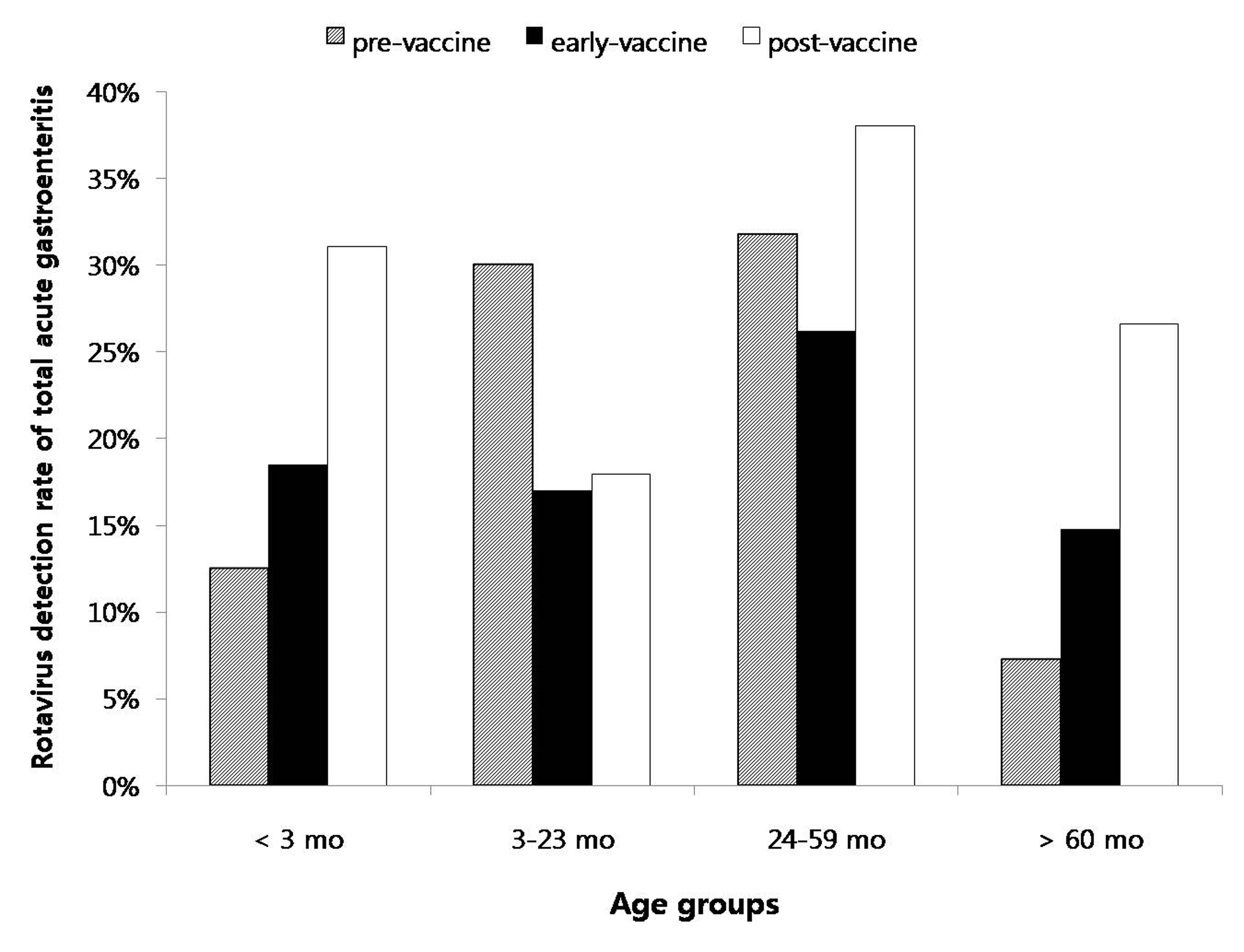

Supplement: Additional file 3: Figure S3. — Changes of the rotavirus infection rates by ages during pre-vaccine (2004–2006), early-vaccine (2008–2010), and post-vaccine (2011–2015) periods. (JPG 1221 kb) [file 12879_2016_1623_MOESM3_ESM.jpg]
