# Supplementary material for: Changing distribution of age, clinical severity, and genotypes of rotavirus gastroenteritis in hospitalized children after the introduction of vaccination: a single center study in Seoul between 2011 and 2014
Source: BMC Infect Dis. 2016 Jun 14;16:287. doi: 10.1186/s12879-016-1623-y (PMC4906974; doi:10.1186/s12879-016-1623-y)
Supplement: Additional file 4: Table S1. — Distribution of group A rotavirus G and P genotypes according to vaccination status, n (%) (DOCX 45 kb) [file 12879_2016_1623_MOESM4_ESM.docx]

Supplemental Table S1. Distribution of group A rotavirus G and P genotypes according to vaccination status, n (%)

|  | Vaccinated with Rotateq | Vaccinated with Rotarix | Incompletely vaccinated | Unvaccinated cases | Total | p-value* |
| --- | --- | --- | --- | --- | --- | --- |
|  | n=14 | n=5 | n=6 | n=154 |  |  |
| Common genotypes | | | | | |  |
| G2P[4] | 1 (7.1) | 2 (40.0) | 0 | 30 (19.5) | 33 (18.4) | 1.000 |
| G1P[8] | 2 | 0 | 3 (50.0) | 21 (13.6) | 26 (14.5) | 1.000 |
| G3P[8] | 0 | 0 | 0 | 9 (5.8) | 9 (5.0) | 0.365 |
| G9P[8] | 0 | 0 | 0 | 5 (3.2) | 5 (2.8) | 0.096 |
| G4P[6] | 0 | 0 | 0 | 1 | 1 (0.6) |  |
| G4P[8] | 0 | 0 | 0 | 1 | 1 (0.6) |  |
| subtotal | 3 (21.4) | 2 (40.0) | 3 (50.0) | 67 (43.5) | 75 (41.9) | 0.229 |
| Uncommon genotypes | | | | | |  |
| G3P[4] | 0 | 0 | 0 | 19 (12.3) | 19 (10.6) | 0.138 |
| G1P[4] | 3 (21.4) | 1 | 1 | 13 (8.4) | 18 (10.1) | 0.117 |
| G9P[4] | 1 | 0 | 0 | 6 (3.9) | 7 (3.9) | 0.087 |
| G1P[6] | 1 | 0 | 0 | 1 | 2 (1.1) |  |
| G2P[8] | 0 | 0 | 0 | 1 | 1 (0.6) |  |
| subtotal | 5 (35.7) | 1 (20.0) | 1 (16.7) | 40 (26.0) | 47 (26.3) | 0.489 |
| Multiple genotypes | | | | | |  |
| G1P[4]P[8] | 1 | 0 | 0 | 23 (14.9) | 24 (13.4) | 0.473 |
| G1G2P[4] | 1 | 0 | 1 | 5 (32.5) | 7 | 0.499 |
| G9P[4]P[8] | 1 | 1 | 0 | 2 (12.99) | 4 (2.2) |  |
| G3P[4]P[8] | 0 | 0 | 0 | 4 (2.6) | 4 (2.2) | 1.000 |
| G1G4P[6] | 0 | 0 | 1 | 2 (12.99) | 3 (1.7) |  |
| G1G3P[4]P[8] | 0 | 0 | 0 | 2 (12.99) | 2 (1.1) |  |
| G1G9P[8] | 1 | 0 | 0 | 1 | 2 (1.1) |  |
| G1G3P[8] | 1 | 0 | 0 | 0 | 1 (0.6) |  |
| G1G4P[8] | 0 | 0 | 0 | 1 | 1 (0.6) |  |
| G2G3P[4] | 0 | 0 | 0 | 1 | 1 (0.6) |  |
| G2P[4]P[8] | 0 | 0 | 0 | 2 (12.99) | 2 |  |
| subtotal | 5 (35.7) | 1 (20.0) | 2 (33.3) | 43 (27.9) | 51 (28.5) | 0.377 |
| nontypeable | | | | | |  |
| none | 1 | 0 | 0 | 2 (2.6) | 3 (1.7) |  |
| P[4] | 0 | 0 | 0 | 2 (2.6) | 2 (1.1) |  |
| G9 | 0 | 1 | 0 | 0 | 1 (0.6) |  |
| subtotal | 1 | 1(1.3) | 0 | 4 (2.2) | 6 (3.4) | 0.131 |
| Total | 14 (100) | 5 (100) | 6 (100) | 154 (100) | 179 (100) |  |

*vaccinated vs unvaccinated
