# Supplementary material for: Changing distribution of age, clinical severity, and genotypes of rotavirus gastroenteritis in hospitalized children after the introduction of vaccination: a single center study in Seoul between 2011 and 2014
Source: BMC Infect Dis. 2016 Jun 14;16:287. doi: 10.1186/s12879-016-1623-y (PMC4906974; doi:10.1186/s12879-016-1623-y)
Supplement: Additional file 5: Table S2. — Distribution of group A rotavirus G and P genotypes according to the vaccination status (exclude incomplete vaccination cases, n = 173) (DOCX 34 kb) [file 12879_2016_1623_MOESM5_ESM.docx]

Supplement Table S2. Distribution of group A rotavirus G and P genotypes according to the vaccination status (exclude incomplete vaccination cases, n=173)

|  | Vaccinated case (%) | Unvaccinated case (%) | Total case | p value |
| --- | --- | --- | --- | --- |
|  | n = 19 | n = 154 | n=173 |  |
| G type* | 18 | 150 | 168 |  |
| G1 | 11 (61.1) | 69 (46.0) | 80 | 0.225 |
| G2 | 4 (21.2) | 39 (26.0) | 43 | 0.729 |
| G3 | 1 (5.6) | 34 (22.7) | 35 | 0.126 |
| G4 | 0 (0) | 5 (3.3) | 5 | 1.000 |
| G9 | 5 (27.8) | 14 (9.3) | 19 | 0.020 |
| P type^+^ | 17 | 152 | 169 |  |
| P[4] | 11(41.2) | 105 (69.1) | 116 | 0.712 |
| P[6] | 1 (5.9) | 5 (3.3) | 6 | 0.476 |
| P[8] | 7 (36.8) | 71 (46.1) | 78 | 0.664 |
| multiple G, P or G/P type^++^ |  |  |  |  |
| G1G2 | 1/18 (5.3) | 5/150 (3.2) | 6/168 | 0.584 |
| P[4]P[8] | 3/18 (15.8) | 32/152 (20.8) | 35/170 | 0.663 |
| G1P[4]P[8] | 1/18 (5.3) | 23/152 (15.1) | 24/170 | 0.473 |
| multiple GP | 6/17(31.6) | 42/150 (27.3) | 48/167 | 0.529 |
| Untypeable | 19 | 154 | 173 |  |
| G untypeable | 1 (5.3) | 4 (2.6) | 5 | 0.445 |
| P untypeable | 2 (10.5) | 2 (1.3) | 4 | 0.060 |
| G or P untypeable | 2 (10.5) | 4 (2.6) | 3 | 0.131 |

*: Total number of G type cases excluded G untypeable ones

^+^: Total number of P type cases excluded P untypeable ones

^++^: G or P untypeable cases for which the specific G/P combined genotype could not be determined were excluded.
